# Eliminating Algorithmic Racial Bias in Clinical Decision Support Algorithms: Use Cases from the Veterans Health Administration

**DOI:** 10.1089/heq.2023.0037

**Published:** 2023-11-30

**Authors:** Justin M. List, Paul Palevsky, Suzanne Tamang, Susan Crowley, David Au, William C. Yarbrough, Amol S. Navathe, Craig Kreisler, Ravi B. Parikh, Jessica Wang-Rodriguez, J. Stacey Klutts, Paul Conlin, Leonard Pogach, Esther Meerwijk, Ernest Moy

**Affiliations:** ^1^ VA Office of Health Equity, Washington, District of Columbia, USA.; ^2^ Department of Internal Medicine, Yale School of Medicine, New Haven, Connecticut, USA.; ^3^ Kidney Medicine Section, Medical Service, VA Pittsburgh Healthcare System, Pittsburgh, Pennsylvania, USA.; ^4^ Renal-Electrolyte Division, Department of Medicine, University of Pittsburgh School of Medicine, Pittsburgh, Pennsylvania, USA.; ^5^ Department of Veterans Affairs, Palo Alto, California, USA.; ^6^ Department of Medicine, Stanford University School of Medicine, Stanford, California, USA.; ^7^ Nephrology Section, Department of Medicine, Yale School of Medicine, New Haven, Connecticut, USA.; ^8^ VA Connecticut Healthcare System, West Haven, Connecticut, USA.; ^9^ Health Services Research and Development, VA Puget Sound Health Care System, Seattle, Washington, USA.; ^10^ Department of Internal Medicine, The University of Texas Southwestern Medical Center, Dallas, Texas, USA.; ^11^ VA North Texas Health Care System, Dallas, Texas, USA.; ^12^ Corporal Michael J. Crescenz VA Medical Center, Philadelphia, Pennsylvania, USA.; ^13^ Department of Medical Ethics and Health Policy, Perelman School of Medicine, University of Pennsylvania, Philadelphia, Pennsylvania, USA.; ^14^ Analytics and Performance Integration (API), Office of Quality and Patient Safety, Veterans Health Administration, Washington, District of Columbia, USA.; ^15^ Perelman School of Medicine, University of Pennsylvania, Philadelphia, Pennsylvania, USA.; ^16^ VA National Pathology and Laboratory Medicine Service, Washington, District of Columbia, USA.; ^17^ Department of Pathology, University of California San Diego School of Medicine, La Jolla, California, USA.; ^18^ National VHA Diagnostics Office, Washington, District of Columbia, USA.; ^19^ Iowa City VA Healthcare System, Iowa City, Iowa, USA.; ^20^ Department of Pathology, University of Iowa Carver College of Medicine, Iowa City, Iowa, USA.; ^21^ VA Boston Healthcare System, Boston, Massachusetts, USA.; ^22^ Department of Medicine, Harvard Medical School, Boston, Massachusetts, USA.; ^23^ Department of Veterans Affairs, New Jersey Health Care System, East Orange, New Jersey, USA.

**Keywords:** Veterans Health Administration, racial bias, predictive models, clinical equations, health equity

## Abstract

The Veterans Health Administration uses equity- and evidence-based principles to examine, correct, and eliminate use of potentially biased clinical equations and predictive models. We discuss the processes, successes, challenges, and next steps in four examples. We detail elimination of the race modifier for estimated kidney function and discuss steps to achieve more equitable pulmonary function testing measurement. We detail the use of equity lenses in two predictive clinical modeling tools: Stratification Tool for Opioid Risk Mitigation (STORM) and Care Assessment Need (CAN) predictive models. We conclude with consideration of ways to advance racial health equity in clinical decision support algorithms.

## Introduction

The use of race-adjusted clinical equations and algorithms spans many medical specialties and often has historical roots pointing to racism in medicine. Race adjustment has been shown as harmful in multiple clinical scenarios.^1,2^ Race, a social construct, is used in both clinical medicine and health services research. As a proxy for structural racism in health-related research, race and ethnicity potentially identify health disparities, in turn provoking further examination of the health impacts of systemic inequalities and social determinants. When relied on as a proxy for generalizing biological differences, race proves to be imprecise for delineating genetic and phenotypic variance.^3^

Furthermore, the conflation of ancestry and race creates clinical risks, where race is used instead of available genotype and phenotype testing to guide treatment decisions. For example, researchers found that the immunosuppressant, azathioprine—an important drug option for treating conditions such as systemic lupus erythematosus—was discontinued or the dose was reduced unnecessarily for some Black patients based on abnormal cell count laboratory values using reference ranges standardized for largely White populations.^4^

When accounting for a known genotype and the presence of the Duffy null phenotype (associated with a lower baseline absolute neutrophil count than found in laboratory result reference ranges), these patients may have safely stayed on azathioprine. The researchers proposed more widespread phenotype testing for certain conditions since the related genotype, while more common in people of African ancestry, can be found in other ancestral populations.

Similarly, clinical decision support tools using machine learning are also at risk of introducing or exacerbating racial bias.^5^ Health care systems increasingly use clinical decision support tools to gauge potential health outcome risk among patients. Many of these tools use predictive modeling to assess risk. While they are potentially powerful tools to capture patients at increased risk for morbidity and mortality, they are also at risk of perpetuating health inequities by introducing unintended racial bias.

As the largest integrated health care system in the United States, the Veterans Health Administration (VA) serves over 9 million enrolled veterans and provides a wide range of care services across nearly 1300 health care facilities.^6^ The VA has begun addressing algorithmic racial bias and promoting equitable clinical equations^7^ and decision support tools as part of larger health equity initiatives.

The VA established the Office of Health Equity (OHE) in 2012 and assembled the Health Equity Coalition, which together created the Health Equity Action Plan (HEAP).^8^ As a living document, the HEAP strategically guides equity work across five aims: awareness; leadership; health outcomes; workforce cultural and linguistic competency; and data, research, and evaluation.

Currently, >20% of veterans come from minoritized racial/ethnic backgrounds, and the OHE works with partners to publish actionable reports that identify disparities across many demographic characteristics in quality of care and veteran patient experience.^9^ VA patients have some of the lowest mortality disparities nationally,^10^ but as a learning system, VA continues to actively monitor and identify racial/ethnic and other disparities that persist.

For example, the periodically produced National Veteran Health Equity Report describes racial/ethnic, geographic, gender, and age disparities in veteran patient experience and quality of care for conditions such as hypertension, hyperlipidemia, and diabetes.^9^ In addition, VA specialty care programs, such as the VA Kidney Medicine Program, track multiple health equity-related demographics in the delivery of specialty care as strategic program goals.

The work of advancing equity belongs to all VA staff, and content-specific efforts are led across VA national program offices and facilities. In this article, we present four use cases from across VA program offices that address racial bias in clinical equations or decision support algorithms. Authors from the OHE reviewed an inventory of clinical equations with known bias^1^ and identified two case studies where there was knowledge of clinical leaders advocating for and pursuing changes to equations. We selected two cases focusing on decision support algorithms based on knowledge of existing work to mitigate racial bias and the widespread use and impact of these tools. We discuss approaches, successes, challenges, and next steps in addressing and eliminating racial bias both in these examples and more broadly.

## Estimated Glomerular Filtration Rate

Assessment of kidney function includes calculation of an estimated glomerular filtration rate (eGFR) using laboratory and demographic variables. Reductions in the eGFR may signal the presence of kidney disease and are central to the diagnosis and staging of chronic kidney disease (CKD), a condition that has affected an estimated 37 million (15%) Americans and 1 million enrolled veterans.^11,12^

Many equations exist for calculating the eGFR, such as the widely utilized Modification of Diet in Renal Disease Study and the 2009 Chronic Kidney Disease Epidemiology Collaboration (CKD-EPI), both of which include a race coefficient for Black individuals based on epidemiologic data.^1^ As a result, these equations risk overestimating kidney function in some Black individuals, delaying the recognition of CKD and implementation of a disease-modifying therapy, as well as referral for kidney transplantation.^13^ Additionally, researchers and others began questioning the inclusion of race based on serum creatinine observational data, muscle mass, or other factors.^14^

In 2020, the National Kidney Foundation (NKF) and the American Society of Nephrology (ASN) established a task force to address the inclusion of race in the diagnosis of kidney disease, and in September 2021, a new CKD-EPI eGFR equation that did not include race was released.^15^ The NKF/ASN Task Force published its report calling for immediate implementation of the new equation.^16^ The VA played an active role in this process: VA clinician researchers both led and participated in the NKF/ASN Task Force that recommended against using race-based eGFR equations.^17^

The VA National Kidney Medicine Program and the VA National Pathology and Laboratory Medicine Programs developed a collaborative, national strategic plan to implement the race-neutral equation. The VA Pittsburgh Medical Center was the first VA facility to implement the new equation within 30 days of electronic publication of the new race-neutral 2021 CKD-EPI eGFR equation from the task force report, and all VA laboratories were mandated to implement the race-neutral calculation by April 1, 2022.^7^

As part of an equity mindset and ensuring clinical excellence, VA has been at the vanguard of implementation of cystatin C testing, providing an alternative race-neutral assessment of kidney function, and continues to monitor the impact of moving to the 2021 CKD-EPI eGFR calculation.^18–20^ VA leadership collaborates with clinicians to incorporate all clinical data and individual patient factors where eGFR is used as an input in clinical decision-making.

Impact analysis of implementation of the race-free eGFR equation has shown no difference in the estimated number of veterans impacted by CKD, but did show geographic shifts to higher CKD prevalence in regions with higher numbers of Black veterans and an increased number of Black veterans with CKD stages 3 and 4.^20^ Such analyses permit advance preparation to reallocate resources accordingly and ensure equitable access to specialty care in the future.

Identifying CKD at the earliest stage possible is essential for preventing or delaying progression to kidney failure requiring dialysis or transplantation. In July 2022, the Organ Procurement and Transplant Network (OPTN) Board implemented a policy prohibiting transplant programs from using race-based eGFR calculations in determining eligibility for transplant. Subsequently, in December 2022, the OPTN Board adopted a policy that by January 3, 2024, all kidney transplant programs have to identify Black transplant candidates whose original qualifying date was affected by the race-based eGFR calculation.^21^

Specifically, transplant centers are required to (1) determine if the use of the race-neutral 2021 CKD-EPI equation would have resulted in qualifying earlier and gaining waiting time for a transplant; (2) apply to the OPTN to modify the waiting time for eligible candidates; (3) contact and inform currently registered candidates for kidney transplant about this initiative; (4) contact candidates a second time to let them know if they qualify for a waiting time adjustment; and (5) submit attestation of compliance to the OPTN.

As of the time of this article, all VA Transplant Centers have implemented the race-neutral eGFR calculation and are on track to complete the eGFR recalculation, updation of waiting times, and veteran outreach, as required by the OPTN policy.

## Pulmonary Function Testing

Evaluation of lung health includes pulmonary function tests (PFTs), the components of which include spirometry, measuring lung volume, maximum respiratory pressure, and diffusion capacity. Interpretations of PFT measurements are used to diagnose a wide array of lung diseases and, as a result, they impact the timeliness and use of potential treatments. Interpretation of PFTs affects treatment plans for other conditions such as bone marrow transplant and the use and dosage of chemotherapy. Additionally, PFT interpretation may directly or indirectly affect eligibility for certain types of employment (e.g., firefighters), disability benefits, and insurance premiums.

The expected values derived from spirometry testing incorporate a patient's age, height, sex, and race/ethnicity compared with a nonsmoking, healthy, and racially/ethnically concordant representative population. The origins of including race/ethnicity in spirometry calculations are mired with racialized assumptions about innate lung function differences compared with White individuals rather than more plausible explanatory mechanisms such as exposure to childhood environmental pollution, nutrition and health disparities, and gene–environment interactions.^22–24^

Race/ethnicity coefficients in spirometry calculations were not designed as, and are poor proxies for, structural racism and other causal factors that affect lung function. For some racial/ethnic groups, the use of race-specific equations may result in differential classification of the severity of lung disease, with implications for access to certain disease treatments and disability benefits.^24^ However, at the same time, these racial corrections have allowed treatment options (both surgical and medical) that may have been otherwise excluded based on lung function.

The widely used 2019 American Thoracic (ATS)/European Respiratory Society guidelines include both race/ethnicity-specific and ethnicity-neutral (e.g., “other/mixed,” race-neutral) options.^25^ The ongoing use of race-specific equations continues to risk exacerbating health inequities for some individuals. Conversely, universally switching to the current race-neutral equation may widen the limit of normal function for some groups and narrow it for others, resulting in missed diagnoses in some individuals and overtesting and overdiagnosis in others.^24^

In April 2023, the ATS—acknowledging ongoing concerns about using race-specific equations as well as concerns about ignoring race—recommended universal movement to a race-neutral equation and called for a broader reevaluation of how PFTs are used to make clinical, employment, and insurance decisions.^26^

In light of this recommendation, clinicians continue to navigate an imperfect state of the science and the nuanced considerations for using either race-neutral coefficients universally or continuing with race/ethnicity-specific ones. Patients at the threshold of the lower limits of normal in PFT interpretation particularly need careful consideration of all factors that go into disease diagnosis due to the ongoing imprecision of PFT equation measurements and their interpretation.

The VA National Program Office for Critical Care, Pulmonary, and Lung Cancer Screening is taking a multistep approach to assess the global impact of the new recommendation, including conducting a data analysis of VA-wide data, comparing population measurements using race-specific versus race-neutral equations, and engaging veteran opinion through structured focus groups to better understand how changing to a race-neutral equation may impact multiple racially and ethnically diverse groups of veterans.

The data analysis includes analyzing the possibility that the current race-neutral equation may create new disparities as unintended consequences of the change. While these steps are underway to evaluate PFT equation changes across the entire VA, some individual VA medical centers have decided at local leadership levels to move toward using the race-neutral equation universally while concomitantly collecting race-specific equation data for ongoing analysis of how this change impacts their diverse local patient populations.

Data from the VA-wide analysis, insights from veteran focus groups, and equation implementation considerations will be shared with key internal VA stakeholders as part of formulating the next steps.

## Stratification Tool for Opioid Risk Mitigation

The VA developed the Stratification Tool for Opioid Risk Mitigation (STORM) in 2016 and it was paired with a policy-mandated interdisciplinary case review for very high-risk patients on March 4, 2018.^27^ As a clinical decision support tool, STORM integrates clinical practice guideline-recommended risk mitigation strategies for opioid use disorder, nonpharmacological pain treatment options, and predictive modeling. Using a wide array of demographic, medical, and psychiatric data, the predictive model generates risk scores for patients prescribed opioids.^28,29^ Although demographic factors such as gender and age are included in STORM, currently race and ethnicity are not included.

Recognizing the importance of equity as a foundational principle for real-world clinical decision support, the VA Office of Mental Health and Suicide Prevention's Program Evaluation Resource Center (PERC) began work on development of a framework to assess performance differences in STORM in 2019 based on gender, age, race/ethnicity, and deidentified data.

The framework was developed with a group of stakeholders from VA, Food and Drug Administration's Office of Minority Health and Health Equity, and the Data Science for Social Good program. The stakeholders functioned as subject matter experts for the design of the framework and included clinicians, data scientists, and statisticians.

The framework for algorithmic bias assessment incorporated summary statistics and visual diagnostic tools to help make the information conveyed more intuitive to a diverse set of stakeholders and was customized to the preferences of the stakeholder group. When applied to observational data from 4,214,579 patients enrolled in VA services in 2014, to predict outcomes in 2015, the framework found evidence of performance differences by race/ethnicity.^30–32^

Potential algorithmic bias was detected by differences in false negatives and false positives for each true positive predicted by STORM, plus differences in false omission rates, that is, the rate that a patient predicted to be negative for the outcomes experienced the outcomes. Since this work was performed on deidentified data that lacked some variables used in the version of STORM that was deployed in clinical practice, the analysis used VA data that predated the shift in poor opioid-related outcomes to U.S. citizens of all sociodemographic strata.

The PERC team encountered challenges interpreting results for subgroups of relatively small sizes (e.g., underrepresented marginalized groups such as American Indian/Alaska Native and Asian groups) or subgroups based on more than one demographic factor (e.g., female and >65 years old or female and Black). As a result, PERC decided to extend its work to more recent prediction cohorts.

Building on this work, PERC is applying its performance evaluation framework to 2018 and 2019 outcomes. The framework will also be used to compare alternative techniques for mitigating algorithmic bias, for example, exploring race-specific models or defining race-specific high-risk cut points. While the STORM model does not incorporate race/ethnicity variables, the performance evaluation framework considers race to evaluate potential bias in the STORM model.

Using the PERC framework to monitor performance over time could address model bias as part of continuous process improvement versus an episodic tangential activity. It could also help the STORM modeling team empirically inform when a model should be recalibrated or updated to include a new predictor variable or could benefit from a different prediction approach.

## Care Assessment Need

Care Assessment Need (CAN) is a set of predictive models that aid VA primary care clinical teams in identifying veterans at risk of hospitalization and mortality. Currently, CAN is a set of six distinct algorithms that model three outcomes, hospitalization, death, and hospitalization or death, over two prediction periods, 90 days and 1 year. Predictor variables include demographics, vital signs, medical conditions, prior use of VA health services, laboratory results, and dispensed medications. CAN models are updated weekly and are available to clinicians in population health management tools and dashboards.

Among the general VA population, the CAN models accurately predict outcomes and have demonstrated favorable model discrimination and calibration.^33–35^ Race and ethnicity demographic variables are not included in the CAN model; however, racial/ethnic disparities may emerge through other mechanisms. For example, a not yet published analysis may indicate that Black veterans are systematically underidentified as high risk for 1-year mortality outcomes compared with White veterans.^36^

In an ongoing analysis of the model, age is the most influential variable in the 1-year mortality CAN model. Black veterans die earlier than White veterans and because of variation in average age of mortality, Black veterans may be potentially disadvantaged by the current model, in that it may underestimate their mortality risk, while overpredicting the risk for White veterans.

A VA-funded research team in collaboration with VA operations is exploring statistical concepts of weighting, interaction, penalization, and normalization to make age race-neutral as a predictor variable in an updated model so that CAN becomes more equitable in estimating risk for the model's outcomes.^36^

In addition to exploring the impact of race/ethnicity in the model, the research team is investigating the impact of social determinants of health (SDOH) and weathering, a phenomenon related to the health consequences of experiencing racism,^37^ as reasons for younger average age at death among Black veterans, with the goal of updating the current CAN model to eliminate risk calculation disparities.

Researchers unintentionally discovered that age may be a proxy for weathering, which they continue to analyze. SDOH data elements derived from the electronic health record (specifically, ICD-10-CM SDOH-related Z codes), location-based area statistics, and third-party datasets are being tested for inclusion in CAN.

Both STORM and CAN do not explicitly include race/ethnicity in their predictive models. The performance evaluation framework being used to evaluate STORM includes taking race-specific evaluation approaches to see if algorithmic bias may nonetheless be present through other mechanisms, as discussed here.

In 2020, the Agency for Healthcare Research and Quality issued a request for information (RFI) seeking to gather public and stakeholder perspectives about the efforts and repercussions of addressing algorithmic bias. Many themes emerged in a qualitative study based on the RFI, including the (in)appropriate use of including race and SDOH information in algorithms.

A subtheme included that *when looking to identify risk groups*, (1) race/ethnicity could improve an algorithm in some cases; (2) failure to include race might lead to biased care; and (3) including race/ethnicity might improve an algorithm's predictive accuracy without addressing inequities.^38^ Themes also included acknowledging underrepresentation of racial and ethnic minority groups in datasets, which is not easily remedied by analytic strategies to overcome the insufficient amount of diverse demographic data.

Determining if race/ethnicity variables in algorithms exacerbate bias, versus if, instead, they meaningfully identify and address racial inequities, remains ongoing work for VA researchers and data scientists alongside the entire health care data science community.

## Discussion

VA has begun and seeks to advance health equity, including by examining clinical decision support algorithms, such as clinical equations and predictive models. VA helped lead the national medical society efforts to eliminate the race coefficient from the eGFR calculation and mandated the use of the race-neutral eGFR across its national integrated health care system.

Accelerated by and contemporaneous with evolving PFT guidelines, VA is currently engaging veteran voices and analyzing its national data, looking at the clinical and nonclinical implications of using a variety of approaches for PFT assessment among diverse veteran groups. VA researchers identified racial bias in predictive models such as STORM and CAN, despite race/ethnicity variables being absent, and teams continue to revise modeling to mitigate bias and advance equity.

We also identified challenges, limitations, and areas for improvement when considering the impact and presence of algorithmic bias. The authors may be unaware of existing VA work on other biased equations not discussed here, and an area for improvement includes continuing to systematically identify other biased equations in use, their clinical stakeholders, and any progress on addressing racial bias in them. Similarly, the authors may be unaware of other champions of change to clinical algorithms not discussed in this article, and an area for improvement includes pathways to identify those champions and empower them.

One challenge for advancing the work of eliminating or mitigating algorithmic bias uniformly includes how clinical service lines are organized and their governance structure for identifying and implementing changes to clinical guidance. Not all specialty areas have centralized governance and not all areas that do can change clinical practice guidelines in the ways discussed in this article for the eGFR and PFTs, which themselves used different processes based on the nature of the algorithmic bias, implementation differences, and service line governance structures.

Equation changes that do take place often require clinical champions to identify and advocate for resources for this work, that is, the VA system often relies on clinical service line champions due to governance in a complex health care organizational matrix. National medical society guidelines are crucial levers for pushing large health care organizations, such as VA, forward as well.

Other stakeholders, such as the OHE, can then help advance efforts in eliminating algorithmic bias, but they themselves rely on service lines for creating policy changes. The authors continue to look for ways to identify other equations and decision support tools for potential bias and mitigation.

Another area for improvement includes exploring more deeply the impact of biased algorithms on affected veterans by adapting action- and person-centered elements in frameworks such as the Health ARC (acknowledge, redress, closure), a care delivery model for addressing health inequities.^39^

As an example, the implementation of OPTN requirements for VA kidney transplant candidates affected by the race-based eGFR equation reflects some elements of delivering care in a way that seeks to address prior harms. Across the health system, opportunities remain for creating more equitable care delivery models, including veteran voices that inform steps taken to acknowledge bias and prevent future harm.

We recommend that health care systems look for and consider potential racial bias in equations and predictive models, even where race/ethnicity is not formally included in underlying variable sets. Using a mindset of both curiosity and equity, asking if diagnostic test result interpretation guidelines might be worsening disparities is essential for undoing the legacy of race-based medicine. Equity champions should also consider that corrective actions to address racial bias in equations and predictive models do not inadvertently create bias for other groups or harm patients.

Finally, health care leaders can support advancing equity by empowering internal champions with resources to analyze system data for racial bias. Uncovering racial bias is the first step to advancing racial equity.

## Abbreviations Used

ARC: acknowledge, redress, and closure
ASN: American Society of Nephrology
ATS: American Thoracic Society
CAN: Care Assessment Need
CKD: chronic kidney disease
CKD-EPI: Chronic Kidney Disease Epidemiology Collaboration
eGFR: estimated glomerular filtration rate
HEAP: Health Equity Action Plan
ICD-10-CM: *International Classification of Diseases, 10th Revision, Clinical Modification*
NKF: National Kidney Foundation
OHE: Office of Health Equity
OPTN: Organ Procurement and Transplant Network
PERC: Program Evaluation Resource Center
PFT: pulmonary function test
RFI: request for information
SDOH: social determinants of health
STORM: Stratification Tool for Opioid Risk Mitigation
VA: Veterans Health Administration
